# Living myocardial slices: Advancing arrhythmia research

**DOI:** 10.3389/fphys.2023.1076261

**Published:** 2023-01-13

**Authors:** Jorik H. Amesz, Lu Zhang, Bian R. Everts, Natasja M. S. De Groot, Yannick J. H. J. Taverne

**Affiliations:** ^1^ Translational Cardiothoracic Surgery Research Lab, Lowlands Institute for Bioelectric Medicine, Department of Cardiothoracic Surgery, Erasmus University Medical Center, Rotterdam, Netherlands; ^2^ Translational Electrophysiology, Lowlands Institute for Bioelectric Medicine, Department of Cardiology, Erasmus University Medical Center, Rotterdam, Netherlands

**Keywords:** living myocardial slices, 3D cardiac models, cardiac arrhythmia models, *in-vitro* electrophysiology, translational research

## Abstract

Living myocardial slices (LMS) are ultrathin (150–400 µm) sections of intact myocardium that can be used as a comprehensive model for cardiac arrhythmia research. The recent introduction of biomimetic electromechanical cultivation chambers enables long-term cultivation and easy control of living myocardial slices culture conditions. The aim of this review is to present the potential of this biomimetic interface using living myocardial slices in electrophysiological studies outlining advantages, disadvantages and future perspectives of the model. Furthermore, different electrophysiological techniques and their application on living myocardial slices will be discussed. The developments of living myocardial slices in electrophysiology research will hopefully lead to future breakthroughs in the understanding of cardiac arrhythmia mechanisms and the development of novel therapeutic options.

## Introduction

In order to study electrophysiological phenomena of cardiac arrhythmias and evaluate treatment options, multiple *in-vitro* systems and *in-vivo* models have been developed over the past decades. Historically, the field of electrophysiology has greatly advanced based on animal studies, ranging from studies on *Caenorhabditis elegans* (Fischer et al., 2017) and zebrafish (Wilkinson et al., 2014) to large animals such as pigs and sheep. These animal models are still valuable for specific research questions. Yet, no model recapitulates the complete human interplay between electrophysiology, hemodynamics and cardiac morphology, so it is important to recognize species differences in cardiac action potentials, membrane currents and consequential calcium handling (Blackwell et al., 2022). Off note, animal models using for example pigs or goats are valuable due to their high resemblance to the human setting with similar cardiac size, anatomy and cardiac electrophysiology. However, use of such models is hampered due to increasing global ethical constraints, high costs and time to breed (Blackwell et al., 2022).

This prompted parallel exploration of other types of disease models. Two-dimensional cardiomyocyte cultures dominated the field of electrophysiological research for many years. Nonetheless, those *in-vitro* models fail to fully recapitulate the complex three-dimensional (3D) architecture of the heart despite the advances towards 3D cell cultures. The fact that stem-cell derived cardiomyocytes have relative immaturity in terms of their metabolic, contractile molecular and electrophysiological properties is still a major limitation of the field (Goldfracht et al., 2020). Furthermore, building 3D constructs cannot account for the complex adherence allowing bioelectric cell-cell communication between cells. Hence, there is still a growing demand for dedicated preclinical platforms that have a high degree of human cardiac electrophysiological mimicry.

Attention was therefore redirected to the use of living myocardial slices (LMS). The LMS technique is not new, but short-term cultivation of LMS and consequential interpretation and extrapolation of the results hampered its widespread use. Recent technological advances enabled the transition of LMS towards a more representative model of the human myocardium (Bursac et al., 2003; Camelliti et al., 2011). A major step forward in the myocardial slicing technique was achieved by the introduction of special electromechanical cultivation chambers (Fischer et al., 2019; Watson et al., 2019), enabling long-term cultivation of LMS cultures. The aim of this review is to present the potential of a biomimetic interface using LMS in electrophysiological studies outlining the advantages, disadvantages and future perspectives of the interface.

### Living myocardial slices

LMS are ultrathin (150–400 μm and 6–17 cardiomyocyte layers) sections of cardiac tissue that are directly produced from cardiac specimens, leaving the native 3D microarchitecture and intracellular connections of the heart intact. The thin nature of the slices allows diffusion of oxygen and nutrients into the innermost cell layers, with a maximum diffusion distance of 200 µm from top and bottom surface of the slice (Shattock et al., 1983; Barclay, 2005). LMS have several advantages over cellular *in-vitro* cardiac models, in which the extracellular matrix with complex 3D architecture is lost. Moreover, cellular models often do not display all cell populations present in the heart and connections between cells are poorly expressed, while the *in-vivo* heart is characterized by a complex interplay of different cell types and communication between cells (Perbellini and Thum, 2019). Multicellularity and intercellular contact are important for proper investigation of myocardial propagation of electrical waves and should therefore be present in a representative electrophysiological model of the human heart. LMS maintain the native structure of the heart because they are directly ‘cut’ from intact cardiac tissue with a high-precision vibratome, and therefore present a model with a high degree of *in-vivo* mimicry suitable for electrophysiological research.

### Brief history of myocardial slices in electrophysiological research

**FIGURE 1:**
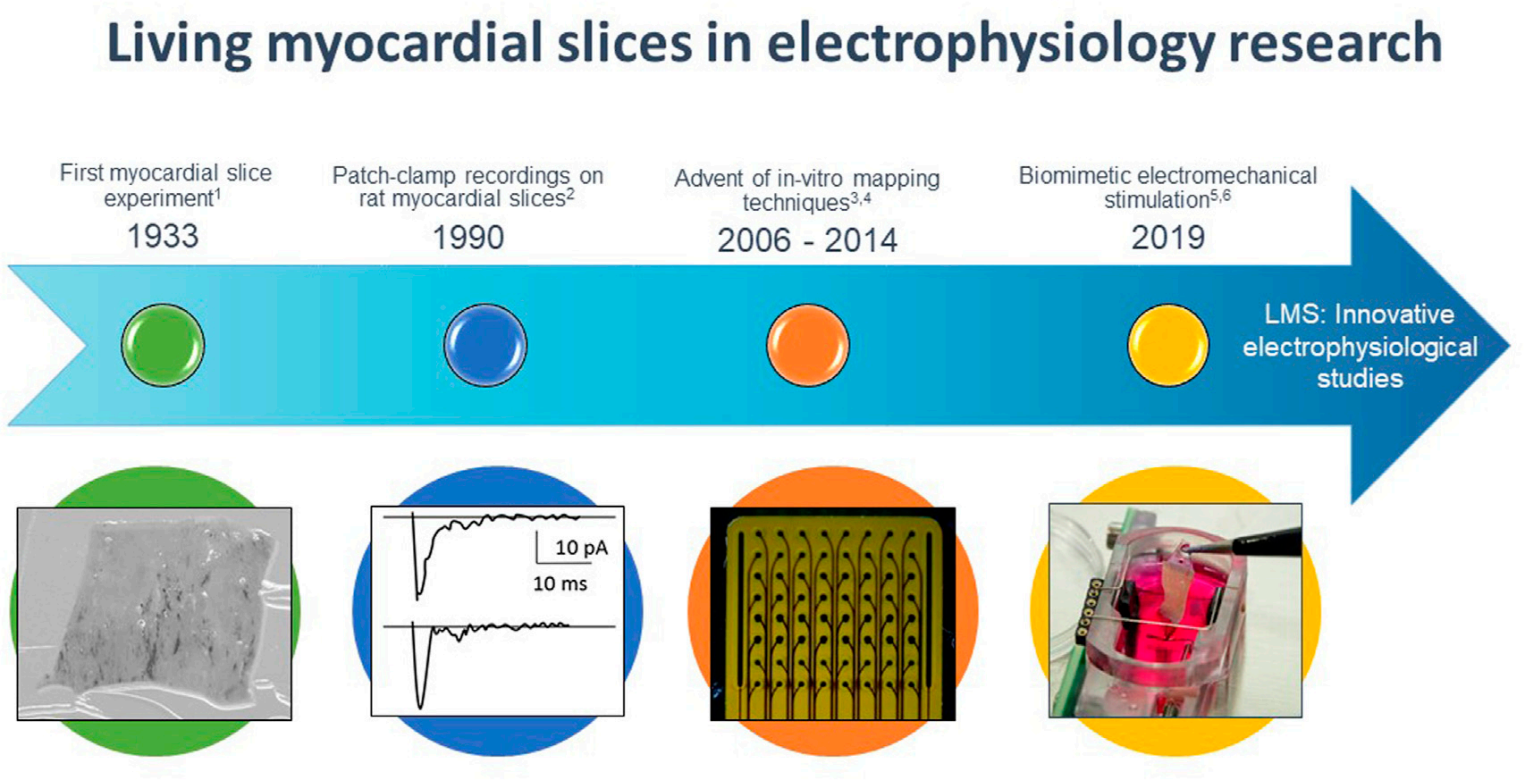
Timeline of major events for living myocardial slices (LMS) in electrophysiological research. 1 Pincus (1933), 2 Burnashev et al. (1990), 3 Halbach et al. (2006), 4 Wang et al. 2015, 5 Fischer et al. (2019), 6 Watson et al. (2019).

The use of myocardial slices dates back to 1933 when Pincus studied the effects of drugs on the oxygen consumption of several excised tissues, including cardiac ventricular tissue (Figure 1) (Pincus, 1933). Burnashev et al. (1990) were the first to use myocardial slices for cardiac electrophysiological research, demonstrating patch clamp recordings on rat cardiac muscle slices. However, broad interest in this technique remained scarce until Bursac et al. (2003) demonstrated that rat myocardial slices possessed more representative electrophysiological characteristics (connexin 43 expression and action potential characteristics) in comparison to cultured cardiomyocytes. From then on, more groups started employing cardiac slices for electrophysiological research, and especially the entrance of microelectrode arrays led to renewed interest in the myocardial slice as an electrophysiological model (Pillekamp et al., 2005; Camelliti et al., 2011). During that same period, experiments with optical mapping of myocardial slices were performed (Kang et al., 2016). The most important breakthrough in LMS culture was achieved in 2019 when two groups developed novel technologies that prevented significant and functional changes in LMS associated with chronic *in-vitro* culture, now allowing for LMS cultivation of several weeks to months (Fischer et al., 2019; Watson et al., 2019). Since then the number of articles published on experiments utilizing myocardial slices increased considerably (Pitoulis et al., 2020b).

### Development of biomimetic cultivation chambers

Before 2011, culturing LMS was limited by the short period of cardiomyoycte viability in culture (**≈**24 h). This was first addressed with the development of culture systems with liquid-air interfaces (Brandenburger et al., 2011; Kang et al., 2016) and continuous electrical stimulation and oxygenation of the culture medium (Ou et al., 2019). As such, LMS cultivation was possible for 6–28 days. However, lack of mechanical loading of these slices still resulted in rapid decline of contractile force and altered cardiac expression *in-vitro*. In 2019, two groups presented novel biomimetic cultivation chambers that provide uniaxial mechanical loading and continuous electrical stimulation to LMS, improving cardiac phenotype and preserving contractile force of LMS (Fischer et al., 2019; Watson et al., 2019). Watson et al. (2019) developed custom-made stretchers for LMS which were placed in culture chambers with continuous flow and oxygenation of the culture medium. Electrical stimulation was performed with field stimulation in between two carbon electrodes. In this manner, isometric twitch forces were generated in the tissue. This resulted in highly functional LMS for 5 days. Fischer et al. (2019) choose a different approach with linear auxotonic loading of slices. Here, LMS were also mounted in a custom-made stretcher, but one end of the stretcher was able to move during contraction, resulting in elastic contractions. The movement of the flexible end of the stretcher was directly correlated to contractile force, allowing real-time measurement of LMS twitch force during culture. These culture chambers were placed on rocking plates for continuous agitation of the culture medium and electrical field stimulation was applied in between two graphite electrodes. As such, a stable beating state of LMS was reached for up to 4 months. However, adaptation in gene expression occurred during the first 8 days of culture, including components of excitation-contraction coupling and the extracellular matrix, and targets of adrenoceptor signalling. Later, Pitoulis et al. (2022) further optimized these static loading platforms by developing a system with dynamic mechanical loading, better simulating electromechanical events of the cardiac cycle with not only preload but also afterload. A 3-Element Windkessel operated in sync with the electrical stimulation to dynamically load LMS with force transducer feedback and a stretcher actuator. This resulted in force-length measurements, corresponding with pressure-volume loops *in-vivo*, and allowed for different (pathological) loading conditions (culture duration: 3 days). Recently, this concept was even taken further by not only providing dynamic loading in one axis, but in a more three-dimensional fashion with a pneumatically driven flexible membrane that induces stretch to the LMS on top of it (culture duration: 12 days) (Miller et al., 2022). Hence, cultivation in biomimetic cultivation chambers with electromechanical loading has proven to improve LMS culture conditions, and the community is still working on novel chambers to facilitate optimal physiological long-term cultures of LMS (Table 1). For electrophysiological research, the next step in the development of LMS cultivation chambers comprises the integration of electrodes and electrophysiological mapping techniques in the chambers.

**TABLE 1:** Overview of different biomimetic cultivation chamber interfaces for myocardial slices.

Biomimetic interface	Type of loading	Oxygenation	Electrical stimulation
Brandenburger et al. (2011)	No loading	Liquid-air interface	No stimulation
Watson et al. (2019)	Isometric 2D	Perfusion	Field
Fischer et al. (2019)	Auxotonic 2D	Agitation on rocker plate	Field
Pitoulis et al. (2022)	Dynamic 2D	Perfusion	Field
Miller et al. (2022)	Dynamic 3D	Daily oxygenated medium exchange	Field

### LMS technique

The technique to produce LMS has been described in detail (Brandenburger et al., 2011; Watson et al., 2017; Fischer et al., 2019; Watson et al., 2019; Watson et al., 2020; Hamers et al., 2022). In this review, we will focus on the technique to produce LMS from human (residual) tissue, which we believe best mimics human (electro-)physiology because of cellular and structural differences between humans and animals. Yet, the technique to produce LMS from small mammals is rather similar (Watson et al., 2017) and has been important for the development of the technique. Moreover, small mammals present with easy access to cardiac tissue material, and the possibility to produce LMS from material of healthy controls.

Human tissue for the production of LMS is obtained from patients undergoing open-heart surgery, such as cardiac transplantations, assist device implantations, atrial appendage amputations, septal myectomies, or different types of congenital surgeries. Specimens are immediately submerged in cold 4°C Tyrode’s solution with the addition of excitation-contraction uncoupler 2,3-butanedione monoxime (BDM), preventing the tissue from contracting during slicing. In the laboratory, excessive endocardial trabeculae and epicardial fat are removed from the cardiac tissue, and the tissue is submerged in 37°C low-melting agarose for structural support on the vibratome. This tissue block is mounted on the vibratome in 4°C Tyrode buffer supplemented with BDM and it is placed with the flat epicardium down to align fibers in the same plane within the tissue. It is of utmost importance that the myocardial fibre direction of the tissue is longitudinal to the moving direction of the vibratome blade in order to obtain viable slices. Watson et al. (2019) showed 97% viability of cardiomyocytes within slices if these critical steps of fibre orientation and alignment are applied. The vibratome then produces slices with a thickness of 150–400 µm coming from different layers within the myocardium. In general, vibratome speed should be lowered for atrial tissues in comparison with ventricular tissues, as atrial tissue is more fragile and therefore at higher risk for rupturing on the vibratome (Kang et al., 2016; Amesz et al., 2022). In addition, slices infiltrated with large amounts of fibrous and adipose tissue often show separation of myocardial bundles and are therefore not routinely selected for culture.

Next, slices are mounted in special cultivation chambers filled with 37°C cell culture medium and superperfused with oxygen or placed on a rocking plate to provide diffusion of oxygen and nutrients to the cardiomyocytes. Medium-199 with insulin-transferrin selenium and antibiotics is the culture medium of choice for most groups working with LMS (Brandenburger et al., 2011; Kang et al., 2016), sometimes supplemented with antioxidants, growth factors and/or hormones (2-mercaptoethanol, fibroblast growth factor, vascular endothelial growth factor, (nor)adrenaline, triiodothyronine, and/or dexamethasone) (Fischer et al., 2019; Ou et al., 2019; Pitoulis et al., 2022). In the cultivation chambers, mechanical preload can be applied in different fashions (Fischer et al., 2019; Watson et al., 2019; Miller et al., 2022; Pitoulis et al., 2022). Electrical field or point stimulation results in cardiac contractions of the LMS, which can be monitored in real-time with dedicated force transducers. Electromechanical stimulation enables long-term cultivation of LMS and offers long-term analyses of LMS biomechanics, biochemistry, histology and electrophysiology.

## Electrophysiological model

As mentioned before, LMS have a high-degree of *in-vivo* mimicry of electrophysiological properties of the human heart because of their intact native tissue structure. Hence, cardiomyocytes in LMS are well-aligned in a striated fashion and linked by gap junctions, and membrane bound connexins are preserved (Habeler et al., 2009). To date, LMS have been predominantly produced from ventricular specimens, but can also be produced from atrial tissue (Girmatsion et al., 2009; Biliczki et al., 2019; Amesz et al., 2022). In addition, Kang et al. (2016) produced slices from the sinoatrial node region and observed automaticity in these LMS. Hence, it could be of interest to produce slices from regions explicitly involved in cardiac arrhythmias, for better understanding of ectopic foci throughout the atria and underlying mechanisms of cardiac arrhythmias (Kang et al., 2016; Li et al., 2022). Next to that, one could produce LMS from patient material with and without history of cardiac arrhythmias, making LMS a disease-specific platform. Furthermore, anatomical differences in electrophysiology between the endocardial and epicardial layers can be addressed (Wen et al., 2018; Pitoulis et al., 2020a). For example, Wen et al. (2018) already showed longer action potential (AP) and calcium transient duration, and more calcium transient alternans in the endocardium compared to the epicardium.

As demonstrated by Pertsov et al. (1993) and Watanabe et al. (2017), LMS could be used to study the role of cardiac re-entry underlying tachyarrhythmias, induced by extrastimulation. This questions the widely accepted theory that re-entry is only possible in hearts exceeding a “critical mass” of more than ≈400 mm^2^ and ≈12 g (Garrey, 1914; Winfree, 1994) as LMS present with a surface area of ≈80 mm^2^. Yet, in order to obtain re-entry in such a small LMS, the refractory period has to be relatively short and/or conduction relatively slow. Next to that, investigating the role of electrical breakthrough waves in the pathophysiology of tachyarrhythmias seems more difficult with a LMS model. In theory, breakthrough waves could occur in LMS (150–400 µm) comprised of multiple (6–17) cell layers if cell layers are activated asynchronously due to the presence of intramural block, leading to dissociation in patterns of activation between the top and bottom layers of the LMS (Kharbanda et al., 2018). Yet, up to date, there has been no report on this phenomenon in LMS. This may be the result of lack of mapping technologies capable of detecting very small local activation time differences between the various layers. If such breakthrough waves would occur in LMS, they could be detected by simultaneous mapping of the top and bottom of the slice, if the sampling frequency of the recording system is high enough (Kharbanda et al., 2018).

**FIGURE 2:**
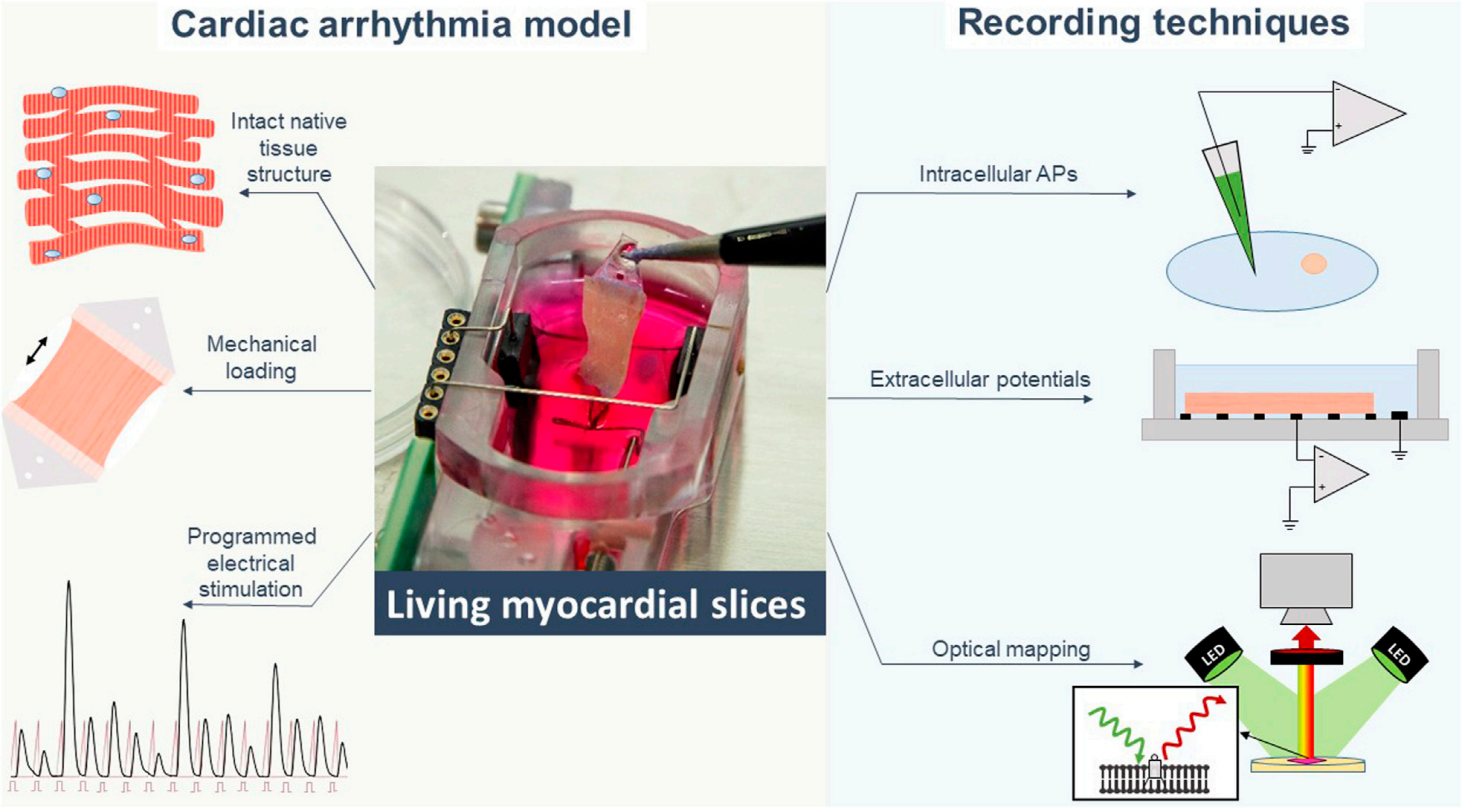
Living myocardial slices are a cardiac arrhythmia model with high degree of *in-vivo* mimicry to which different electrophysiological recording techniques can be applied. APs action potentials.

The development of special biomimetic cultivation chambers also makes LMS a model with precise control of (patho-)physiological conditions. For example, mechanical overload can be induced by the application of extra stretch on myocardial tissue, leading to a fibrotic remodelling response (Nunez-Toldra et al., 2022; Pitoulis et al., 2022) associated with cardiac arrhythmias. At the same time, application of programmed electrical stimulation protocols in the cultivation chambers allows for mimicking arrhythmias, e.g., with programmed rapid electrical stimulation or frequent extrasystoles (Figure 2) (Amesz et al., 2022). Furthermore, viral transduction can be established in LMS (Ferrera et al., 2005; Kang et al., 2016; Thomas et al., 2016) for cardiac disease induction and gene therapy (Liu et al., 2020). Combining gene targeting technologies with mechanical conditioning and programmed electrical stimulation, LMS represent a potent system for the study of disease-specific activity including arrhythmias (Schneider-Warme et al., 2017).

This review mainly focuses on electrophysiological techniques applied to LMS. Yet, LMS also enable the possibility to perform comprehensive studies on the mechanistic understanding of arrhythmias. Fischer et al. (2019) developed a cultivation chamber with real-time measurement of contractile forces, without having to place the LMS on external force transducers. Such a platform can be used for continuous evaluation of contractile responses to different culture conditions, including tachypacing and induction of arrhythmias (Amesz et al., 2022). Moreover, structural and molecular analyses of LMS further supplement electrophysiological and mechanical assessments of LMS. Histology provides analysis of cellular and extracellular matrix organization, and specific biomolecules can be labeled and visualized. In addition, biochemical analyses at the genomic, proteomic and secrotomic level can be easily applied to LMS when snap-frozen after culture (Watson et al., 2020). These analyses can also be conducted on the spent culture medium to evaluate biochemical events in LMS, without termination of LMS cultivation. Although, analyses can be complicated due to the relatively small number of cells in LMS, resulting in low concentrations of secreted molecules in the culture medium. As such, LMS provide a platform with an easy assessment of different parameters using already available kits and techniques from other cardiac *in-vitro* research models. The next section of this review will specifically discuss the application of dedicated electrophysiological techniques on myocardial slices.

### Intracellular action potential recording

The patch clamp technique has been the golden standard for basic electrophysiological experiments since its invention in the 1970s (Neher and Sakmann, 1976). The patch-clamp system is capable of measuring membrane voltages by attaching micron-sized diameter glass pipettes to the cell membrane. The membrane patch under the micropipette tip is broken and the electrode inside the micropipette is electrically connected to the cytoplasm to measure the intracellular transmembrane potential, resulting in an AP.

In 1990, Burnashev et al. (1990) were the first to apply the patch-clamp technique to rat cardiac slices. They reported resting membrane potentials (RMP) between −30 and −65 mV and sodium and inward rectifying potassium currents similar to isolated ventricular cells and proved single-channel recordings in LMS without the need for complicated cell isolation procedures. In addition, this technique showed proof-of-concept and avoided the possible alteration of channel properties caused by proteolytic enzymes during myocardial cell isolation, although, RMP of −30 mV indicate cellular damage to some extent.

Since then, the patch-clamp technique has been used in combination with LMS in a few studies. In 2011, Huang and Li explored the feasibility of whole-cell patch-clamp recordings from cardiac ventricular slices of newborn rats. The sodium current, outward potassium current, hyperpolarization-activated cyclic nucleotide–gated (HCN) channel, and AP all displayed features similar to those previously reported for isolated rat ventricular myocytes (Huang and Li, 2011). Consequently, Nong et al. (2013) performed patch-clamp recordings in ventricular slices from HCN4-gene transfected mice and found altered electrophysiological characteristics consisting of a greater negative amplitude and expression of funny currents.

The sharp electrode technique is an alternative approach for patch-clamping and is more commonly used on LMS. Here, a glass electrode with very small pore diameter is directly inserted in the cell to measure its membrane potential. Intracellular recordings of APs performed with sharp glass electrodes showed a fast upstroke, a high amplitude and short AP duration at 50%, characteristic for murine adult ventricular APs (Himmel et al., 2012), indicating that cardiomyocytes within the LMS were electrophysiologically intact. In 2016, Peinkofer et al. (2016) used this technique to study the AP differences between native murine atrial and ventricular cardiomyocytes from early embryonic development through adulthood. Measurements revealed significant changes in AP morphology between atria and ventricles and pre- and postnatal murine development. Next to that, they investigated the interaction between stem cell-derived cardiomyocytes and ventricular slices and showed electrical integration of the transplanted cardiomyocytes in the recipient slices (Peinkofer et al., 2017; Peinkofer et al., 2021).

For intracellular AP recording in LMS, the sharp electrode technique is more often used compared to the patch clamp technique, because the latter is more complicated in multicellular preparations with high amount of extracellular matrix proteins (Peinkofer et al., 2016). Yet, an advantage of the patch clamp technique is the possibility to record individual ion currents, which is not possible with sharp electrodes because membrane potentials cannot be clamped in the electrically well-coupled syncytium (Peinkofer et al., 2016). Hence, patch-clamps and sharp electrodes both have their perks and drawbacks and the technique of choice for intracellular measurements of APs in LMS depends on the research question. Additionally, non-invasive optical imaging techniques may provide an alternative solution for the disadvantages of these two techniques.

### Optical mapping

Cardiac optical mapping is a fluorescent imaging technique to record electrical activity and calcium transients in the heart. Measurements are performed with fluorescent dyes of which the emitted light is captured by an imaging system with high spatial and temporal resolution. To date, few studies have used optical mapping in combination with LMS.

Optical mapping allows the measurement of a multitude of parameters, including membrane potential with voltage sensitive dyes (VSDs) and intracellular free calcium ([Ca^2+^]_i_) dynamics with calcium dyes. Fast VSDs are often based on a styryl component (Efimov et al., 2004) and enable the recording of optical APs. In addition, AP duration, activation, conduction velocity, and repolarization maps can be acquired based on changes in fluorescence (O'Shea et al., 2020; Efimov et al., 2004; Kang et al., 2016; Berenfeld and Efimov, 2019). Based on these parameters, AP upstroke analysis, arrhythmia analysis, and phase mapping can be performed (Efimov et al., 2004; Laughner et al., 2012).

Calcium dyes are used to visualize spatial changes in the kinetics and amplitude of calcium transients (Del Nido et al., 1998; Laurita et al., 2003; Venkataraman et al., 2012). The binding of a high-affinity calcium dye to [Ca^2+^]_i_ can affect calcium handling by prolonging the calcium transient (Herron et al., 2012). Therefore, low-affinity dyes, such as Fura-4F, are preferred (Wokosin et al., 2004; Fast, 2005). Calcium dynamics are usually displayed in colour-coded maps (Jaimes et al., 2016) which are used to interpret the mechanisms of arrhythmias (Davidenko et al., 1992; Omichi et al., 2004; Warren et al., 2007) and heart failure (Lang et al., 2015). The calcium upstroke and extrusion phases, and calcium transient time to peak are used to study the activity of cellular exchangers, pumps and ryanodine receptors (RyR). Intracellular calcium transient duration is a Ca^2+^ analogue of the AP duration in optical mapping (Jaimes et al., 2016). Furthermore, calcium dyes are used to study calcium kinetics that influence contractility. A combination of VSDs and calcium dyes gives insights into excitation-contraction coupling and arrhythmia mechanisms (Jaimes et al., 2016).

The main advantages of optical mapping over currently available electrode measurements are the high spatial resolution, the ability to simultaneously measure several physiological parameters, and the absence of artefacts due to electrical stimulation (Efimov et al., 2004; Wang et al., 2015; Berenfeld and Efimov, 2019). Optical mapping also has several limitations and factors to consider. Firstly, optical mapping presents with the risk of illumination-induced phototoxicity caused by the production of reactive oxygen species (ROS) (Schaffer et al., 1994). This limits the study time for optical mapping and possibility to perform repeated measurements over time. However, phototoxicity is less pronounced in multicellular aggregates and tissue preparations, compared to single cells (Kanaporis et al., 2012). Moreover, recent developments in fluorescent dyes have resulted in dyes that can be excited at longer, red-shifted wavelengths, greatly reducing the necessary illumination intensity, which in turn reduces the risk of ROS-production (Matiukas et al., 2007). Secondly, a major limitation of optical mapping is the presence of motion artefacts caused by contraction. Commonly, these motion artefacts are suppressed with electromechanical uncouplers such as blebbistatin and BDM. However, this may result in alterations in AP duration and calcium handling (Kappadan et al., 2020). Another solution is the use of motion tracking algorithms in combination with ratiometric imaging, in which the ratio between two separate excitation or emission wavelengths is taken to attenuate noise (Bachtel et al., 2011; Kappadan et al., 2020). Thirdly, a complete optical mapping system can be very expensive, with prices of $40.000 for a CCD or CMOS camera. Finally, there has not been a consensus on the depth of field in optical mapping yet. However, ranges from 300–500 μm (Knisley, 1995; Girouard et al., 1996) and ranges up to 2 mm (Baxter et al., 2001) have been reported. In either case, it can be assumed that sufficient fluorescence can be measured from LMS with a thickness of **≈**300 μm. It should be noted that every cell layer in a LMS contributes to the emitted fluorescence signal. Hence, the recorded fluorescence should be interpreted as an averaged signal throughout the slice thickness.

In the early 90s, optical mapping on cardiac slices was pioneered by the group of Jose Jalife et al. (Davidenko et al., 1990; Davidenko et al., 1992; Pertsov et al., 1993; Cabo et al., 1994; Davidenko et al., 1995). They showed initiation of sustained re-entrant excitation in small ventricular slices, which provided a major contribution to the understanding of spiral waves leading to re-entrant ventricular tachycardias (Davidenko et al., 1990; Davidenko et al., 1992; Pertsov et al., 1993; Davidenko et al., 1995). Now, optical mapping can be used for a variety of research questions and interventions. For example, Wang et al. (2014) showed the effects of axial stretch on action potential and calcium transient duration in LMS. Watanabe et al. (2017) investigated optogenetic manipulation of anatomical re-entry in ventricular LMS and showed effective termination by induction of local conduction block. And, Miller et al. (2020) and Li et al. (2022) showed cardiotoxicity of cancer drugs with use of optical mapping. Hence, cardiac optical mapping can be successfully performed on LMS for high-quality basic and translational research.

### Extracellular field potential mapping

Cardiac extracellular potential mapping is an electrophysiological imaging technique to record electrical activity of cardiac tissue using multi-electrode arrays (MEAs). MEAs in contact with cardiac tissue record electrical activation waves propagating through the myocardium. The detected electrical signal results from the action potentials of cardiomyocytes located in the vicinity of the electrode (Sim et al., 2019). From these extracellular potentials, multiple parameters can be derived, including peak-to-peak amplitudes, potential duration and slope, and degree of fractionation. Comparison of local activation times between neighbouring electrodes provides information on conduction velocity and hence conduction disorders in the myocardial tissue (de Groot et al., 2022). Such electrophysiological mapping is performed in the clinical electrophysiology lab with bipolar catheters for detection of rhythm disorders and guidance of ablative therapies. Yet, MEAs applied for mapping can also be performed *in-vitro* for monitoring of electrical activity of cultured cardiomyocytes, and also LMS (Reppel et al., 2004).

The most commonly used MEA in LMS research consists of a 60-channel electrode array system with gold contacts (30 μm diameter) arranged in an 8 × 8 electrode grid with an inter-electrode distance range of 200 μm (Multi Channel Systems MCS GmbH, Reutlingen, Germany). This MEA was first used to record extracellular potentials of mouse ventricular slices, demonstrating conduction velocities of 21–24 cm/s (Pillekamp et al., 2005; Halbach et al., 2006; Pillekamp et al., 2006). Halbach et al. (2006) showed that LMS are (electro-)physiologically intact by measuring both APs and extracellular potentials at different stimulation frequencies and with the application of ion channel blockers. In addition. Himmel et al. (2012) demonstrated similar electrical signal characteristics of LMS and other established *in-vitro* and animal models. As such, MEAs can be used for a variety of research questions and interventions. For example, the electrical integration of stem cells in myocardial tissue as cardiac regenerative therapy has been assessed using MEAs and LMS (Hannes et al., 2008; Pillekamp et al., 2009; Rubach et al., 2014). MEAs showed synchronized beating between transplanted stem cells and host LMS, hence demonstrating electrical coupling between both cell types (Hannes et al., 2008; Pillekamp et al., 2009). In addition, conduction block, induced with heptanol administration, could be visualized with colour-coded activation maps constructed with the use of MEAs (Pillekamp et al., 2009).

The simplicity of the MEA allows electrophysiological measurements to be performed without the need for sophisticated skills in complex electrophysiological techniques such as patch clamp (Spach et al., 1972; Chowdhury et al., 2018), or high computational power required for optical mapping signal analysis. The major contributor to successful extracellular potential mapping with MEAs on LMS is the contact between the myocardial tissue and the electrode. Furthermore, the density of the electrodes in the MEA determines its resolution, with a higher resolution providing more accurate information on cardiac conduction. Although the resolution of MEAs has improved over recent years with developments in MEA technology, it still presents with lower resolution as compared to optical mapping measurements (van der Does and De Groot, 2017; Hansen et al., 2018). Another factor to consider is the possible separation of myocardial bundles in LMS, reducing the representation of the *in-vivo* electrical activation patterns by LMS, especially if the tissue is infiltrated with fibrous and adipose tissue. Integrity of the slices can be improved if proper vibratome settings are applied, but data interpretation in these regions should be done carefully. Altogether, electrophysiological properties of LMS were verified using MEA techniques, promoting and broadening the potential of this technology for pharmacological drug testing and performing studies under (patho)physiological conditions (Bussek et al., 2009).

### Current state and future perspectives

Most electrophysiological studies in this review are of methodological nature and describe intrinsic electrophysiological characteristics of LMS and techniques to reliably measure these features. These reports have shown that LMS present a valid platform with close *in-vivo* (electro)physiological resemblance, which is important for reliable interpretation and extrapolation of the results. Yet, as validity of the LMS model has been confirmed, we encourage the electrophysiological community to (further) incorporate this technique in her laboratories, in order to unravel detailed mechanistic properties of arrhythmias and test novel anti-arrhythmic therapies based on these insights. For example, Esfandyari et al. (2022) identified microRNAs that regulate the cardiac action potential and demonstrated that manipulation of these microRNAs in LMS can affect action potential duration and refractoriness. And, Watanabe et al. (2017) showed optogenetic manipulation of anatomical re-entry in ventricular LMS, being a potential therapeutic option for termination of reentrant tachyarrhythmias. These are examples of innovative therapies for cardiac arrhythmias, based on technological advances in cardiac cell biology and electrophysiology, which were tested in LMS.

The next step in LMS research should be the incorporation of dedicated mapping techniques into the biomimetic cultivation chambers. To date, optical and extracellular potential mapping has only been performed on unloaded slices, being less representative of the *in-vivo* volume loading of the heart which influences electrophysiological characteristics. Moreover, the incorporation of electrophysiological measurements in biomimetic cultivation chambers allows for direct correlation between electrical signal characteristics and force measurements (Fischer et al., 2019; Watson et al., 2019) to investigate excitation-contraction coupling mechanisms of cardiac arrhythmias (Grandi et al., 2012). The addition of biochemical and histological analyses to these force and electrical measurements makes LMS a platform capable of correlating different types of biological data coming from a single specimen with high-degree of *in-vivo* mimicry. As such, LMS will become an even more important player in the field of basic cardiac electrophysiology research.

## Summary

LMS have been around for a few decades, but widespread use within the electrophysiological research community has been hampered due to functional degradation of LMS in culture. The introduction of biomimetic electromechanical cultivation chambers, enabled long-term cultivation and easy control of culture conditions of LMS. LMS provide a model with high-degree of human *in-vitro* electrophysiology mimicry, due to their native structure with conduction proteins and intact cell-cell interactions. Feasibility of intracellular AP and extracellular FP recording techniques has now been demonstrated extensively, showing similar conduction and potential characteristics to *in-vivo* and other *in-vitro* modalities. As such, LMS provide a valid model for mimicry of cardiac arrhythmias, and will hopefully lead to future breakthroughs in the understanding of cardiac arrhythmia mechanisms and development of novel therapeutic options.
